# Advancements in exosome-based cancer diagnosis: from chipsets to nano vaccine

**DOI:** 10.1080/15384047.2025.2541991

**Published:** 2025-08-03

**Authors:** Nobendu Mukerjee, Subham Sarkar, Daniel Ejim Uti, Prashant Kumar Sharma

**Affiliations:** a Centre for Infectious Diseases & Microbiology, School of Public Health Sciences and Technology, Hyderabad, India; b Centre for Global Health Research, Saveetha Institute of Medical and Technical Sciences, Saveetha University, Chennai, India; c Department of Biochemistry/Research and Publications, Kampala International University, Kampala, Uganda; d Department of Biochemistry, Faculty of Basic Medical Sciences, College of Medicine, Federal University of Health Sciences, Otukpo, Nigeria; e RFIC Bio Research Centre, Kwangwoon University, Seoul, South Korea; f Department of Life Sciences, Imperial College London, London, UK

**Keywords:** Exosomes, cancer nano-vaccines, lab-on-a-chip, tumor heterogeneity, immune system interaction, nanotechnology

## Abstract

Exosome-based therapies represent a pioneering frontier in cancer treatment, leveraging the natural cellular communication mechanisms encapsulated in exosomes. These nano-sized vesicles serve as carriers of proteins, lipids, and nucleic acids, reflecting the physiological state of their cells of origin, which makes them ideal candidates for targeted cancer therapies and diagnostics. Despite their potential, the path to clinical application is fraught with challenges. This review explores the inherent challenges associated with exosome-based cancer vaccines, focusing on tumor heterogeneity, the technical difficulties in exosome isolation and characterization, the need for standardized protocols, and the scalability of production methods. It also explores the interaction between exosomes and the immune system, a crucial factor in developing effective cancer vaccines. The review explores strategies to improve diagnostic tools, targeted delivery systems, and therapy based on individual tumor profiles, highlighting the need for innovative approaches and collaborative efforts to maximize exosome-based cancer vaccines’ therapeutic potential

## GRAPHICAL ABSTRACT

**Figure:**
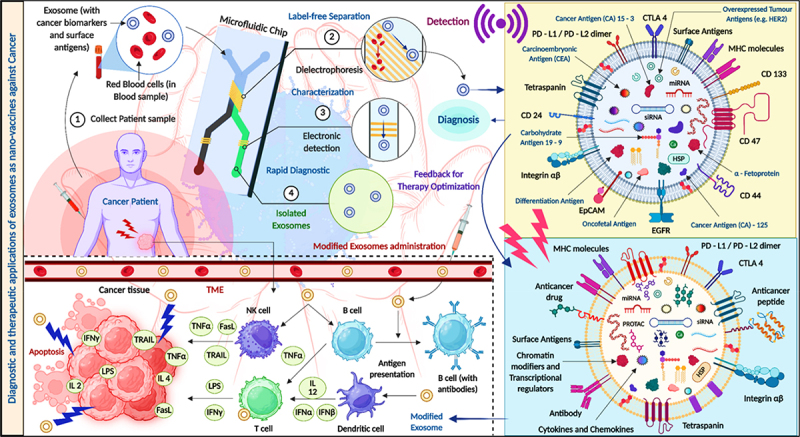

## Introduction

Exosomes, vesicles released by every cell type, have drawn attention in biomedical research and therapeutic applications, especially to treat cancer. Ranging from 30 to 150 nanometers in diameter, these nano-sized vesicles have been found to be vital for intercellular communication by delivering a plethora of molecular cargos, e.g. proteins, lipids, RNA, and even DNAs to far-away destination sites from their biological point of departure. This capacity to delivery biological cargo has propelled exosomes into the center stage of innovative therapies, specifically vaccine development and diagnostic applications for treating cancer.^1^

Extracellular vehicles (EVs), have additional roles in metastasis apart from intercellular communication in promoting pre-metastatic niche formation and as organ sites for metastasis establishment by either stimulating or repressing non-cancerous cells,^2^ while at the same time regulating anti-cancer immune responses.

Cancer is still one of the top leading causes of death all over the world, and traditional forms of treatment, like chemotherapy, radiation, and surgery, are often not able to provide a solution due to their systemic toxicity and non-selectivity. This creates an urgent need to identify more targeted and less invasive therapeutic strategies, such as nano vaccines. Nano vaccines use nanotechnology and target a much stronger and specific immune response against cancer while exerting smaller side effects on healthy tissue.^2^ Exosome-based nano vaccines represent one of the newest applications of the concept “nano vaccine,” by utilizing exosomes that the body naturally produces to stimulate immunity. Traditional vaccines typically use inactivated disease-causing organisms, or synthetic substances, which would likely cause an immune response. Exosomes exhibit excellent characteristics for a vehicle, including biocompatibility, stability in the bloodstream, and natural intercellular communication. If exosomes could be engineered to incorporate specific antigens associated with cancer cells, exosomes could signal the immune system to mount an appropriate immune response at the exact location the immune response is needed, effectively “teaching” the immune system to recognize and attack cancer cells.^3^

**Figure 1.**
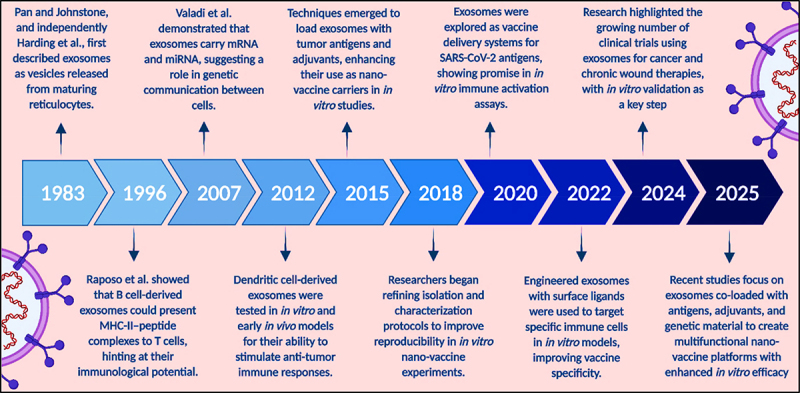
Pipeline of development to EVs in research (created with BioRender.com).

Exosome-based nano vaccines differ from traditional vaccines in regard to their delivery mechanisms and specificity. Traditional vaccines often deliver an antigen in a nonspecific manner, prompting the immune system to recognize and react to that particular antigen in the body. This may result in unwanted side effects and an inadequate immune response. Conversely, exosome-based nano vaccines deliver antigens specifically to relevant immune cells, typically dendritic cells, which are crucial for activating the immune system. This targeted delivery improves the immune response, reduces the likelihood of side effects, increasing patient safety.^4^ In addition, exosomes can carry multiple types of cancer-specific antigens, a potentially larger immunological attack against many cancer cell lines. Such an individualized approach would be groundbreaking; it would allow for a change in thinking from traditional vaccine immunizations to personalized medicine, namely in the realm of oncology.^5^ Exosome-based nano vaccines represent a way to utilize the body’s natural systems in innovative ways to fight disease. Using the exosomes properties, researchers are unlocking more effective, safe and targeted cancer therapies. This new frontier points to the complexities of cellular communication while welcoming new territory in the endeavor to eliminate cancer altogether^6^ (Figure 1).

**Figure 2.**
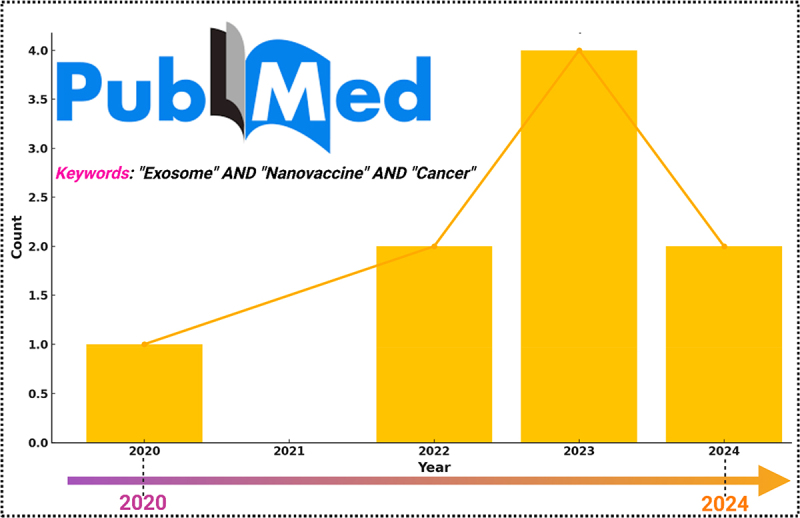
Bibliometric analysis of publication trend on PubMed for the keywords “exosome” and “nanovaccine” and “cancer” from 2020 to 2024.

A comprehensive bibliometric analysis clearly revealed the increasing trend of leveraging exosomes as nanovaccines against cancer in recent years, because of their extraordinary diagnostic and therapeutic potential. This trend is further supported by year-wise publication data retrieved from PubMed using the keywords “Exosome” AND “Nanovaccine” AND “Cancer,” which, although limited in number, show a rising research interest – most notably with a peak in 2023—highlighting the growing focus on this innovative therapeutic approach (Figure 2).

## Exosome biogenesis

Exosome biogenesis is a complex and well-regulated process involving the formation, loading, and secretion of small EVs that serve as a mechanism of intercellular communication by transferring proteins, lipids, and nucleic acids from one cell to another. Understanding exosome biogenesis not only uncovers the mechanisms involved in vital cellular processes, but it also represents a therapeutic means for disease related challenging inventions, diagnostic useful tools, and targeted strategies such as “nano vaccines.” Exosome biogenesis begins in the endosomal system of a cell. The early endosomes, formed by the internal budding of the plasma membrane, will mature and transition to late endosomal compartments when they travel further into the cytosol. In the process of this maturation, some of these late endosomes turn into multivesicular bodies (MVBs) that contain many small vesicles, intraluminal vesicles (ILVs).^7–13^ The ESCRT machinery is the molecular machinery required for ILV production.

**Figure 3.**
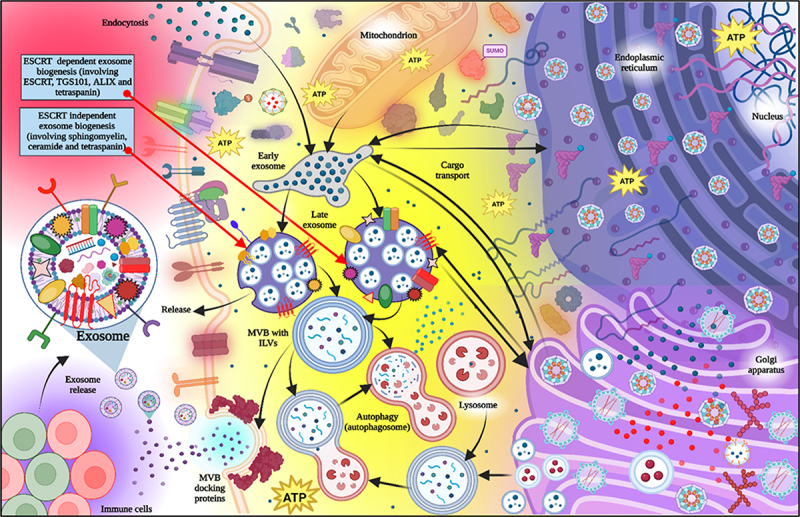
Exosome biogenesis pathway (created with BioRender.com) [modified from ref.].^17^

This complex consists of a range of different protein complexes (ESCRT-0, ESCRT-I, ESCRT-II, and ESCRT-III) which sequentially sort ubiquitinated proteins (and some other proteins) into regions of the endosomal membrane that will bud inward.^8,14^ The ESCRT-III complex has a major role in the budding process since it is responsible for the scission of the membrane to make ILVs within the MVB lumen. In the process of making ILVs, the ILVs are also selectively loaded with a range of different cargoes, including proteins, mRNA, miRNA, and other bioactive molecules. The loading of these different classes of cargo was likely a selective process, determining the ultimate function of the exosomes. There are proteins such as tetraspanins, and heat shock proteins, and other proteins usually associated with exosomal membranes that presumably play roles in cargo selection and vesicle formation. In addition to the roles of the proteins, some specificity of the cargoes may be mediated by lipid-related events, including ceramide (derived from the breakdown of sphingomyelin by neutral sphingomyelinase), which was recently demonstrated to facilitate ILVs formation independent to the ESCRT machinery.^9,10,15^ The pathway taken by MVBs is likely determined by cellular signals, either they fuse with lysosomes for degradation of their contents, or they migrate to the plasma membrane. The decision as to whether or not to traffic the MVBs toward the plasma membrane to secrete exosomes is dependent on cellular signaling pathways, including those mediated by Rab GTPases, including Rab27a and Rab27b, which play an important role in the docking and fusion of MVBs with the plasma membrane.^11,12,16^ The fusion of the MVBs to the plasma membrane causes the intraluminal vesicles (ILVs) to be released into the interstitial space, with the latter now referred to as exosomes. The release of exosomes in the vesiculation process is dependent on factors including cellular stress, the microenvironment, and the physiological state of the cell (Figure 3).

Exosomes derived from various cell types – such as dendritic cells, tumor cells, and mesenchymal stem cells – exhibit distinct molecular profiles, including variations in their protein, lipid, and RNA cargo. These compositional differences significantly influence their biological function, immunogenicity, and therapeutic applications.^18,19^ Despite this heterogeneity, exosomes consistently express surface markers like CD63, CD81, and TSG101, which are widely used in their characterization and isolation protocols, ensuring reliable identification across studies^20^ (Table 1).

**Table 1.** Comparative overview of exosome sources and their clinical potential.

Exosome Source	Origin	Ease of Isolation	Key Bioactive Contents	Therapeutic Applications	Limitations	Clinical Relevance
Mesenchymal Stem Cells (MSCs)	Bone marrow, adipose tissue, umbilical cord	Moderate to High	miRNAs, cytokines, growth factors (e.g., TGF-β, VEGF)	Wound healing, anti-inflammatory, immunomodulation, tissue regeneration	Batch variability, potential for tumor promotion if not purified	High – Multiple clinical trials underway
Dendritic Cells (DCs)	Peripheral blood or bone marrow-derived precursors	Moderate	MHC I/II, co-stimulatory molecules, miRNAs	Cancer immunotherapy, vaccine delivery	Immunogenicity, scalability issues	Moderate – Used in experimental cancer vaccines
Tumor Cells	Cultured tumor cell lines or patient-derived samples	Moderate	Tumor antigens, oncogenic miRNAs, proteins	Cancer diagnostics, tumor-specific antigen delivery	Risk of promoting tumorigenesis, ethical concerns	Moderate – Diagnostic biomarker potential
Epithelial Cells	Epithelial tissues (e.g., lung, intestine)	Moderate	Cytokeratins, miRNAs, mucins	Biomarker discovery for epithelial cancers, GI diseases	Tissue accessibility, low yield	Emerging – Used in noninvasive diagnostics
Neural Stem Cells (NSCs)	Brain-derived neural precursors	Low	Neurotrophic factors, miRNAs, BDNF, GDNF	Neurological repair (Alzheimer’s, Parkinson’s), stroke recovery	Difficult harvesting, low scalability	Promising – Preclinical studies show neuroprotective effects
Cardiac Progenitor Cells (CPCs)	Heart tissue	Low	Cardioprotective proteins, miRNAs (e.g., miR-21)	Cardiac repair post-myocardial infarction	Low availability, patient-specific variability	Early stage – Limited trials, good preclinical data
Urine-Derived Exosomes	Urine (noninvasive collection)	High	Kidney-specific proteins, miRNAs	Renal disease biomarkers, early cancer detection	Protein degradation risk, dilution issues	High – FDA-cleared assays for some markers
Saliva-Derived Exosomes	Saliva	High	Oral microbiome signals, cancer biomarkers	Oral cancer diagnostics, systemic disease indicators	Contamination, need for standardization	Growing – Noninvasive diagnostics trend
Breast Milk Exosomes	Human breast milk	Moderate	Immunoglobulins, antimicrobial peptides, miRNAs	Infant immunity support, oral drug delivery systems	Ethical sourcing, compositional variability	Moderate – Research in neonatal health applications
Plant-Derived Exosome-like Nanoparticles (PDENs)	Edible plants (e.g., ginger, grapes)	High	Plant miRNAs, polyphenols	Anti-inflammatory, gut microbiota modulation, oral drug delivery	Biological differences with human cells, unclear mechanisms	Emerging – Nutraceutical and oral delivery interest

Factors include hypoxia, oxidative stress, low pH, and nutrient deprivation-environments in which tumor cells often encounter. Many of these factors elevate the rate of exosome secretion, as well as alter the cargo profile, ultimately, leading to exosomes that are enriched for oncogenic proteins and nucleic acids that affect disease progression. The cargo (e.g., miRNAs, MHC molecules) in dendritic cell exosomes differs from the cargo in tumor cell exosomes, which also differs from the cargo in mesenchymal stem cell exosomes. Nonetheless, they typically share common markers including CD9, CD63, CD81, Alix, and Tsg101. Factors include hypoxia, oxidative stress, and acidic pH levels in the environment which vary widely in TME and contribute considerably to exosome secretion and selective cargo profiles.

Exosome biogenesis has important applications in therapeutic strategies, such as exosome-based nano vaccines. Scientists can genetically modify exosomes to contain tumor-derived antigenic material to generate personalized vaccines that prompt a specific immune response against cancer cells using the body’s inherent signaling pathways. This builds on exosomes’ established trajectory as bio-compatible natural delivery vehicles that target cells, thereby optimizing efficacy and minimizing adverse effects. Exosomes typically interact with recipient cells through multiple internalization mechanisms, including clathrin-mediated endocytosis, caveolae-mediated endocytosis, phagocytosis, macropinocytosis, and direct membrane fusion. The internalization mechanism will alter the fate of exosomal content, thereby modifying the downstream metabolic and biological impact.

The involvement of EVs in immune function is significant to the effective design of cancer vaccines. EVs can stimulate or suppress immunity based on their cellular source and their paradoxical molecular contents. EVs produced from dendritic cells or modified tumor cells expressing tumor antigens can enhance antigen presentation and cytotoxic T-cell responses and can represent very strong immunostimulatory agents.^21,22^ Tumor cell-derived secreted EVs generally carry suppressive molecules such as PD-L1, TGF-β, and Fas ligand, which can decrease dendritic cell function, induce regulatory T cells (T_regs_), or induce deletion of T cells, thus allowing for immune evasion.^23,24^ The presence of the duality of EVs is a critical factor in designing cancer vaccines since it emphasizes the need for appropriate donor cells and precise engineering of their cargo to prevent unintended immune suppressive effects. Furthermore, clarifying whether EVs generate immune effects through their influences upon antigen processing pathways, their influence on cytokine release, and cell surface expression of various immune checkpoints is necessary for fully exploiting their vaccine potential.^8,25^ Exosomal uptake can occur through clathrin-mediated endocytosis, macropinocytosis, phagocytosis, and membrane fusion, which can diverge the fate of the cargo and ultimately result in different therapeutic outcomes.

**Figure 4.**
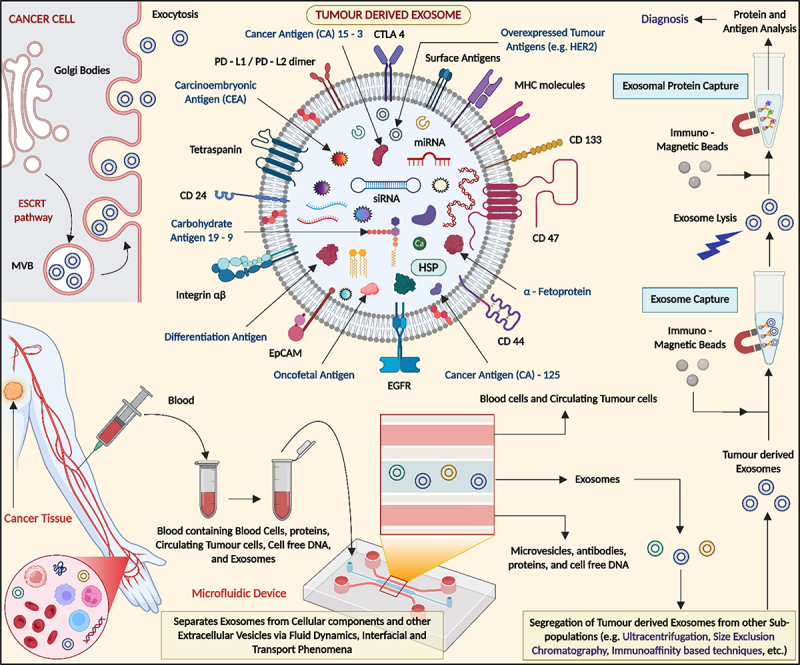
Schematic showing the formation and release of tumor-derived exosomes, their molecular cargo (e.g., proteins, miRnas, tumor antigens), and their isolation from blood using microfluidic and immunoaffinity methods (created with BioRender.com).

Isolation and characterization of exosomes are essential for their clinical and research applications. Common isolation methods include ultracentrifugation, size-exclusion chromatography, ultrafiltration, and immunoaffinity capture. Each technique varies in yield, purity, and scalability. Ultracentrifugation is widely used but may co-isolate non-exosomal particles. Characterization typically involves size and concentration analysis using nanoparticle tracking analysis (NTA) or dynamic light scattering (DLS), along with morphological assessment via transmission electron microscopy (TEM). Surface markers such as CD9, CD63, and CD81 are detected using Western blot or flow cytometry. Cancer-derived exosomes carry diverse oncogenic signatures and biomarkers.^26,27^ Their cargo can be analyzed via chipsets and immunoaffinity techniques for early cancer diagnosis and classification. These steps ensure accurate identification and quality control, which are critical for reliable downstream applications in diagnostics and therapeutics. Figure 4 illustrates the biogenesis, surface markers, and diagnostic isolation methods of tumor-derived exosomes. It highlights the cargo (e.g., miRNA, proteins, tumor antigens) and the separation from blood using microfluidic devices and immunoaffinity techniques for cancer diagnostics.

## Exosome-based nano vaccines

**Figure 5.**
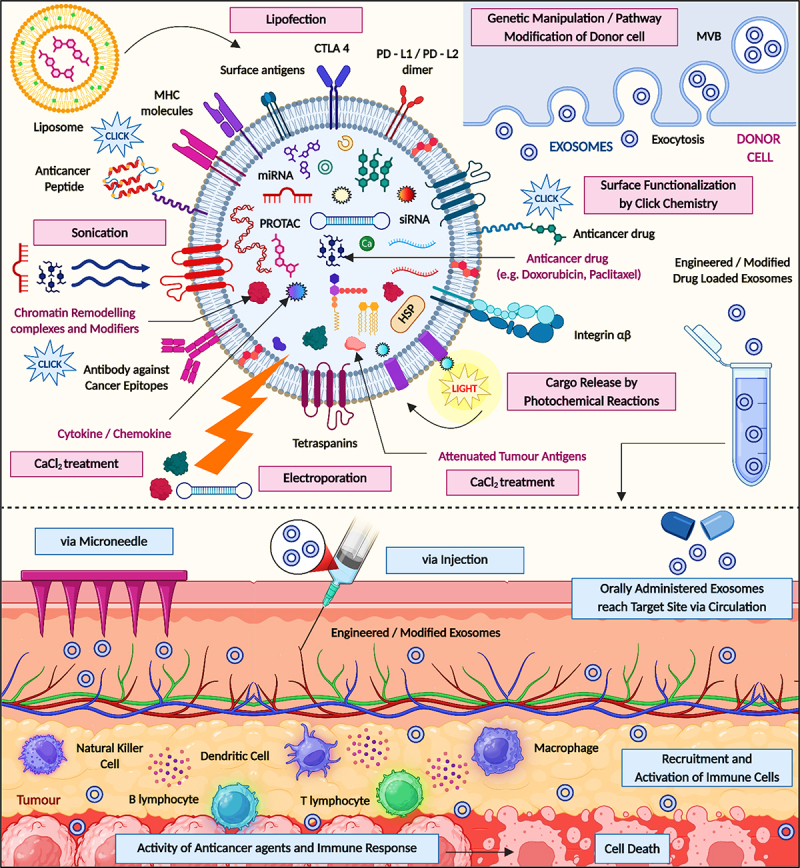
Functionalization strategies of exosomes for cancer therapy, and their prospective administration routes (created with BioRender.com).

Nano vaccines based on exosomes are an advanced technique within immunotherapy, specifically for diseases such as cancer. Nano vaccines utilize the biological characteristics of exosomes to deliver drug agents to target cells. The nano vaccine stimulates an immune response that is specific to treat certain pathologies. The success of nano vaccines is primarily determined by the process of loading the vesicles with vaccine antigens and the distribution technique to the immune system. Exosomes naturally carry and protect a variety of biological material, such as proteins, lipids, and nucleic acids, making exosomes suitable for bio delivery systems. To develop nano vaccines, exosomes can be manipulated to encapsulate specific antigens found in diseases such as cancer. The antigens will be presented to the immune system, which aids in the training of immune cells to identify and kill cells that express the antigens.^28^ The first step in the procedure to develop an exosome based nano vaccine is to determine the source cells that will make the exosomes. As antigen sources, they can be dendritic cells, tumor cells, or even genetically engineered cells to express the antigens of your choice. When the source cells have been identified, they are cultured in a controlled environment to promote exosome production. The exosomes are isolated and purified, usually by differential ultracentrifugation or immune-affinity capture, ensuring the exosome product is free of other cellular debris and contamination.^29^ After an exosome product containing antigens has been isolated, it is injected into the patient, and these antigen-loaded exosomes will be internalized through endocytosis by dendritic cells. Dendritic cells will process the antigens and present them on the cell surface to T-cells, motivating an effective immune response. This pathway of direct antigen presentation is very efficient, as effective activation of both helper and cytotoxic T-cells is possible, which is important for the destruction of cancerous cells or viruses.^30^ This type of cancer vaccine, for which the target is tumor cells, produces a robust and defined immune response against tumor cells, which could lead to clinically-positive patient outcomes (by potentially avoiding the side effects of conventional treatment options like chemotherapy and radiation).^31^ The exosomes customization capabilities allow for personalized medicine. Vaccines can be designed for a given patient’s tumor’s specific antigenic characteristics for a truly tailored treatment. This is very important for high mutation cancers where approaches of therapy have normally failed to control disease. Furthermore, exosome therapeutics, especially nano vaccines, are a hot area of biomedical research that enables the use of the body’s drug delivery system for therapeutics by being utilized as a targeted therapy. As research and technologies advance, exosome-based nanovaccines are poised to significantly influence next-generation disease treatments, particularly by reshaping strategies in vaccine therapy and immunomodulation. The engineering and therapeutic application of modified exosomes for cancer treatment. Exosomes are generated from donor cells through the endosomal pathway and can be engineered using techniques such as electroporation, sonication, CaCl₂ treatment, lipofection, and genetic manipulation to load them with therapeutic agents. These agents include anticancer drugs (e.g., doxorubicin, paclitaxel), siRNA, miRNA, PROTACs, antibodies, peptides, and chromatin-modifying elements. Functionalization methods such as click chemistry and surface ligand modification enhance targeting capabilities. Cargo release can be triggered by external stimuli like photochemical reactions. Functionalized exosomes can be administered through various routes, including intradermal delivery via microneedles, systemic injection (intravenous or intratumoral), and oral administration, depending on the target tissue and therapeutic objective. These routes allow exosomes to circulate and reach specific tumor sites or immune organs efficiently. Upon reaching the tumor microenvironment, engineered exosomes interact with immune cells – such as natural killer (NK) cells, dendritic cells, macrophages, B and T lymphocytes – stimulating an immune response. The result is targeted delivery of therapeutic payloads, activation of immune cells, and induction of tumor cell death. This strategy holds strong potential in precision oncology and immunotherapy by enhancing efficacy while minimizing systemic toxicity (Figure 5). One promising avenue involves the use of EVs to deliver adenoviral vectors as therapeutic vaccines. This approach aims to enhance targeted delivery, reduce immunogenicity, and improve overall efficacy. Exosomes can be modified using various strategies to enable targeted delivery of therapeutic agents. These engineered exosomes are administered into the tumor microenvironment to enhance therapeutic efficacy and reduce off-target effects. The cellular origin of exosomes plays a critical role in their function, especially in vaccine development and immunotherapy. Dendritic cell-derived exosomes contain immunostimulatory molecules such as MHC complexes and co-stimulatory proteins, making them effective in activating adaptive immune responses. In contrast, tumor-derived exosomes, if not properly modified, may promote immune tolerance and support cancer progression. Mesenchymal stem cell-derived exosomes are valued for their immune privilege and regenerative potential. However, their clinical application is limited by issues related to production consistency, batch variability, and standardization. Therefore, both the source of exosomes and the methods used for their modification are key considerations in the development of safe and effective exosome-based therapies.

Antigens can be loaded into exosomes genetically or by incubating exosomes and antigens together so they will naturally encapsulate these molecules. Genetically loading antigens entails manipulating the source organism or cells to express the target antigen with exosomal proteins so that the antigen is loaded into exosomes at the time of formation. Alternatively, we can incubate purified exosomes and antigens together, which will embed antigens into the membrane or within the interior of the exosome depending on its biochemical properties.^30,32^

There are both advantages and disadvantages to the various methods of loading antigens into exosomes. Genetically engineering donor cells to express antigens ensures stable and consistent cargo expression, but the process often requires complex and time-consuming transfection protocols.^33^ Electroporation is another commonly used method for introducing RNA or other molecules into exosomes; however, it carries the risk of vesicle aggregation or membrane disruption, which can compromise functionality.^34^ Passive incubation of exosomes with antigens is technically simple and preserves vesicle structure, but this approach typically yields lower loading efficiency.^35^ Ultimately, the suitability of each method for clinical application depends on reproducibility, safety, and scalability – all of which are crucial for advancing these technologies toward human use^36^ (Table 2).

**Table 2.** Comparison of exosome loading methods for therapeutic applications.

Loading Method	Type	Mechanism	Suitable Cargo	Loading Efficiency	Advantages	Disadvantages	Examples
Incubation (Passive Loading)	Passive	Diffusion of cargo molecules into exosomes by co-incubation under physiological or modified conditions	Small hydrophobic drugs, some RNAs	Low – Moderate	Simple, no equipment needed; preserves exosome structure	Low loading efficiency; limited to lipophilic/small molecules	Paclitaxel, Curcumin-loaded exosomes
Electroporation	Active	Application of electric pulses induces temporary pores in the exosomal membrane to allow entry of cargo	siRNA, miRNA, mRNA, proteins	Moderate – High	Efficient for nucleic acid loading; applicable to various cargos	May cause RNA aggregation or exosome damage; optimization required	siRNA-loaded exosomes for gene silencing
Sonication	Active	Ultrasound disrupts exosomal membrane temporarily, enhancing cargo loading	Small drugs, proteins, siRNA	High	High loading efficiency; suitable for hydrophilic compounds	Potential exosome membrane damage; cargo denaturation possible	Doxorubicin-loaded exosomes
Freeze-Thaw Cycles	Active	Repeated freezing and thawing of exosomes to open and close membranes allowing cargo to diffuse in	Proteins, peptides, nucleic acids	Low – Moderate	Easy to perform; no special equipment required	Aggregation and loss of membrane integrity	Loading catalase for Parkinson’s therapy
Extrusion	Active	Exosome and cargo mixture passed through membranes under pressure causing mechanical loading	Proteins, nucleic acids, nanoparticles	High	High loading; uniform size distribution	May alter exosome structure and functionality	siRNA-loaded exosomes for tumor targeting
Saponin Permeabilization	Active	Use of saponin to transiently permeabilize the membrane to allow entry of cargo	Proteins, enzymes	Moderate – High	Good for protein loading; preserves bioactivity	Saponin toxicity; careful control required	Catalase-loaded exosomes for oxidative stress
Chemical Transfection	Active	Use of commercial transfection reagents to facilitate cargo entry into exosomes	Plasmids, siRNA, DNA	Low – Moderate	Convenient for nucleic acids; available kits	Reagents may be toxic; alters exosome membrane	Exosomes with GFP-plasmids
Endogenous Loading (Cell-engineered)	Biological	Producer cells genetically modified or treated to package therapeutic cargo into exosomes during biogenesis	miRNA, proteins, therapeutic mRNA	High	Physiological loading; maintains exosome integrity	Time-consuming; low scalability; complex optimization	MSC-derived exosomes with anti-inflammatory miRNA
Click Chemistry/Ligand Conjugation	Chemical Modification	Covalent attachment of cargo or ligands to exosomal surfaces or internal groups	Small molecules, peptides, targeting ligands	High	Precise targeting; stable cargo attachment	Complex chemistry; possible immune response	Targeted exosomes with aptamers or peptides

*Direct Loading During Exosome Formation:*

Genetic Engineering: This vehicle involves modifying the genetic structure of the exosome-producing cells so that they express the vaccine antigen along with markers recognized as exosomal. With genetic engineering, the cell can be instructed to package the antigens in exosomes at the exosome formation process. Genomic DNA is introduced into the cell either by transfection with plasmids or transduction with viral vectors to induce the generation a vaccine phrase target for specific cells.^37^

Transfection efficiency: The efficiency of this vehicle is dependent upon successful expression of the antigen in the parent cell and the transporting of the antigen into exosomes as part of the package. The use of promoter sequences to restrict expression of the normal antigen to regions of the cell associated with the exosome production stage can improve specificity and yield of antigen-loaded exosomes.^38^

*Post-Isolation Loading:*

The choice of a suitable loading method is contingent upon the physicochemical properties of the therapeutic cargo and its intended clinical application. Genetic engineering offers high specificity; however, it is technically complex due to the use of viral or non-viral vectors.^39^ Electroporation is commonly used for nucleic acid delivery but can compromise vesicle integrity and result in aggregation.^40^ In contrast, passive incubation is simple and scalable but tends to have lower loading efficiency. Clinical readiness of these approaches also depends on achieving consistency, safety, and compliance with regulatory standards.^36^ Furthermore, the selection of a loading method is influenced by the molecular type: electroporation is often used for RNA, while passive methods are more suitable for peptides and hydrophobic drugs.^41^ Ultimately, the chosen method significantly impacts the yield, cargo retention, and stability of the engineered vesicles.

*Incubation Techniques:*

Once the exosomes are isolated, the exosomes can be incubated with the antigens (as therapeutic cargo) to get these molecules passively loaded to either the exosome membrane or internalized into exosome. This can be done in a co-incubation approach, in which the exosomes and antigens are mixed such that the conditions allow for the inclusion of the antigens into the membranes or internal of the exosome.^42^

*Electroporation:*

Electroporation is another useful approach for loading post-isolation exosomes, creating transient pores in the exosome’s membrane using electric fields and allowing antigens to enter the vesicles. It is worth mentioning that electroporation is a good option for loading of nucleic acids (mRNA or siRNA), since whole genes are the target for expression of the specific antigens once inside the target cells.^43^ The delivery of exosome-based nano vaccines is as important as the loading process. The end goal is to ensure that the loaded exosomes are efficiently taken up by dendritic cells, which are pivotal in the immune system and capable of processing and presenting the antigens to T-cells.

*Targeted Delivery:*

Surface Modification: To improve the targeting efficacy, the surfaces of exosomes can be modified with ligands or antibodies that specifically bind to receptors on dendritic cells. Exosomes can also be developed to express ligands for C-type lectin receptors that are highly present on the surface of dendritic cells, enabling targeted delivery.^44^ Another method for enhancing targeted delivery is the use of homing peptides, which have a natural affinity for specific cell types or tissues. By incorporating these peptides onto the surface of exosomes, it is possible to direct them more precisely to desired target sites. This targeted approach increases the local concentration of therapeutic agents or antigens at the site of interest, thereby enhancing cellular uptake and improving the overall immunogenic response.^45^ Administration of Exosome-Based Nano Vaccines. Common routes for administration of exosome- based nano vaccines is intravenous and subcutaneous injections. These routes administer the exosomes either directly into the blood circulation or deposit them near lymph nodes, which is where dendritic cells are likely to uptake and process the antigens.^46^

Surface modifications such as PEGylation and antibody conjugation significantly influence the pharmacokinetics and therapeutic potential of exosomes. These strategies can extend circulation half-life, reduce immune clearance, and enhance biodistribution and cellular uptake specificity.^47,48^ PEGylation helps exosomes evade recognition by the mononuclear phagocyte system, thereby prolonging systemic exposure. Meanwhile, ligand conjugation enables selective interaction with target cell receptors, improving targeted delivery and minimizing off-target effects.^39^ These surface engineering approaches not only enhance the therapeutic index but also synchronize with upstream strategies such as the use of homing peptides and receptor-specific ligands to ensure more effective delivery of antigens to dendritic cells.

Exosome-based nano vaccines are an innovative approach to cancer treatment by utilizing the biological functions of exosomes and biomedical engineering technology to target cancer cells more appropriately. The technology inherent in the exosome allows it to accurately target only the cancer cells and not the healthy tissue around it. This enhances the efficiency of the vaccines in targeting the cancer cell and provides an advantage over traditional modalities which can cause significant side effects due to their lack of specificity.^31^ The primary benefits of exosome-based nano vaccines are their ability to selectively target tumor cells and their ability to carry variety of different antigenic material. This allows their vaccines to initiate a strong and specific immune response, while reducing the probability the immune system will attack normal body tissues, as many conventional cancer therapies may do.^37^ As exciting as they are, there are challenges to the development of exosome-based nano vaccines such as a need for standardized methods of exosome isolation and loading, ensuring the stability and integrity of the loaded antigens, and overcoming the immune-suppressive environment of tumors that can limit the immune response.^38^ Exosome-based nano vaccines are an exciting prospect for the future of cancer therapy, and a method that may be more specific, effective and less toxic than conventional therapy. Ongoing research and clinical trials are critically important for establishing standardized methods and overcoming other challenges to create the full potential of this new and exciting technology in oncology^42^ (Table 3).

**Table 3.** Clinical trials of exosome based nano vaccine (source: https://clinicaltrials.gov/).

Clinical Trial ID	Cancer Type	Vaccine Description	Phase	Status	Key Objectives	Sponsor
NCT05533697	Advanced Solid Tumors	mRNA-based exosome vaccine	I/II	Recruiting	Evaluate safety, efficacy, and immune response	BioNTech
NCT01550523	Malignant glioma of the brain	Tumor cells	I	Completed	Uses patient’s own tumor cells, treated with antisense molecules to enhance immune response against the tumor	Sidney Kimmel Cancer Center at Thomas Jefferson University
NCT04276441	Melanoma	Dendritic cell-derived exosomes loaded with tumor antigens	I	Active	Assess vaccine’s impact on immune system and tumor response	University Medical Center
NCT03068631	Metastatic Pancreatic Ductal Adenocarcinoma	Mesenchymal stromal cell-derived exosomes	I	Active	Determining maximum tolerated dose and side effects of MSC-derived exosomes loaded with siRNA	M.D. Anderson Cancer Research Center
NCT05559177	Metastatic bladder cancer	Dendritic cells or macrophages	I	Unknown	Evaluates the safety, efficacy, and clinical application of a chimeric exosome vaccine for personalized cancer treatment	Shanghai Pudong Hospital
NCT04389385	Non-Small Cell Lung Cancer (NSCLC)	Exosomes carrying PD-L1 inhibitors	I	Recruiting	Determine safety and preliminary efficacy	Pfizer
NCT01159288	Non-small cell lung cancer	Dendritic cell-derived exosomes (Dex)	II	Completed	Evaluate the efficacy of the vaccine in improving progression-free survival for patients with non-resectable NSCLC	Gustave Roussy, Cancer Campus, Grand Paris
NCT02932956	Prostate Cancer	Exosome vaccine combined with PD-1 blockade therapy	II	Recruiting	Test efficacy in combination with immune checkpoint inhibitors	Merck Sharp & Dohme Corp.
NCT03837764	Various solid tumors	Exosomes as adjuvant to increase immunogenicity	I	Active, not recruiting	Explore optimal dosing and immune activation	National Cancer Institute (NCI)

” >Trials in Table 3 are reorganized by cancer type to aid navigation.

Exosomes can be customized to have multiple inhibitors and antagonists beyond drugs, which all use varying mechanisms to induce cell death such as ferroptosis, block proliferation and control angiogenesis. These inhibitors can also be contained on the surface of the exosomes, in which binding with key receptors on the surface of cancer cells permanently inhibits or limits important downstream signaling pathways for survival and expression of cancer characteristics. Engineering or modifying exosomes is therefore a viable method for targeted therapy against cancer.^49^

### Exosomes as nanovaccines against tumor vascularization and modulators of immune cells

**Figure 6.**
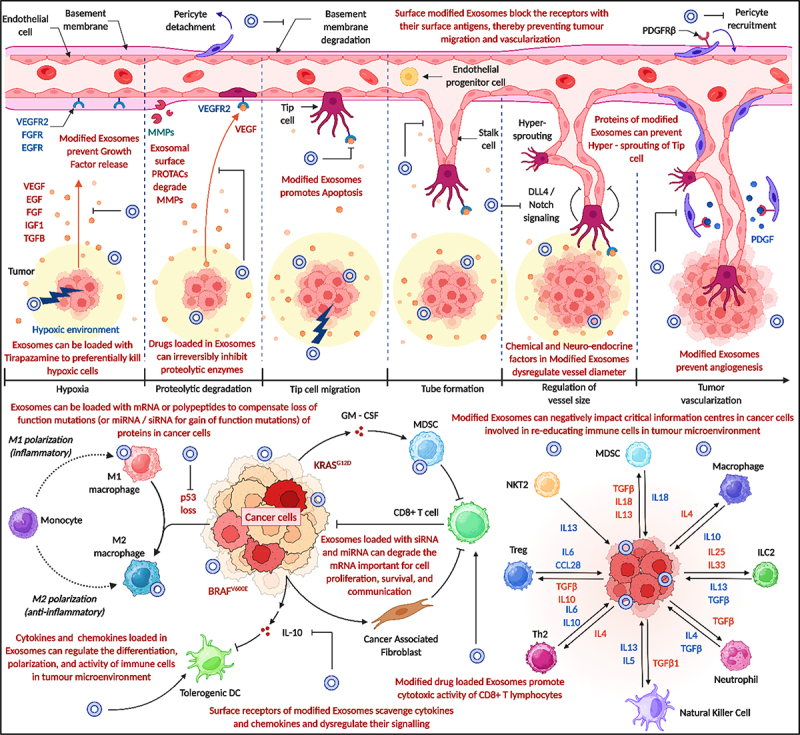
Modified exosomes inhibiting tumor angiogenesis and reprogramming the immune microenvironment. Exosomes block pro-angiogenic signaling and endothelial cell function, while delivering therapeutic cargo to activate immune cells and suppress immunosuppressive populations, enhancing anti-tumor responses (created with BioRender.com).

Exosomes are emerging as effective nanovaccine platforms for targeting tumor vascularization and modulating immune responses. These vesicles can be loaded with tumor-associated antigens, immunostimulatory molecules, and anti-angiogenic factors to induce a strong and specific anti-tumor effect. They promote antigen presentation by dendritic cells, leading to the activation of cytotoxic T lymphocytes and a robust adaptive immune response. Additionally, exosomes can interfere with tumor angiogenesis by delivering molecules that inhibit blood vessel formation, thereby restricting tumor growth and metastasis. They also modulate the tumor microenvironment by enhancing the activity of effector immune cells and reducing immunosuppressive cell populations. Figure 6 illustrates how modified exosomes influence multiple stages of tumor vascularization and immune regulation. They can block receptor-mediated signaling in endothelial cells, prevent growth factor release, and induce apoptosis in tumor-associated vasculature. Additionally, exosomes disrupt tip and stalk cell dynamics through modulation of DLL4/Notch signaling and prevent hyper-sprouting. In the tumor microenvironment, engineered exosomes loaded with siRNA, miRNA, or therapeutic peptides can inhibit oncogenic signaling pathways (e.g., KRAS, BRAF), reprogram macrophage polarization, suppress immunosuppressive cell types like MDSCs and T_regs_, and promote CD8+ T cell activity. This demonstrates their dual role in vascular inhibition and immune reeducation within tumors. Modified exosomes modulate tumor vascularization and influence immune cell behavior. They help reprogram immune cells within the tumor microenvironment to counteract tumor progression.

### Exosome nanovaccines exhibiting anti-TGFβ activity and enhancing immunosurveillance

**Figure 7.**
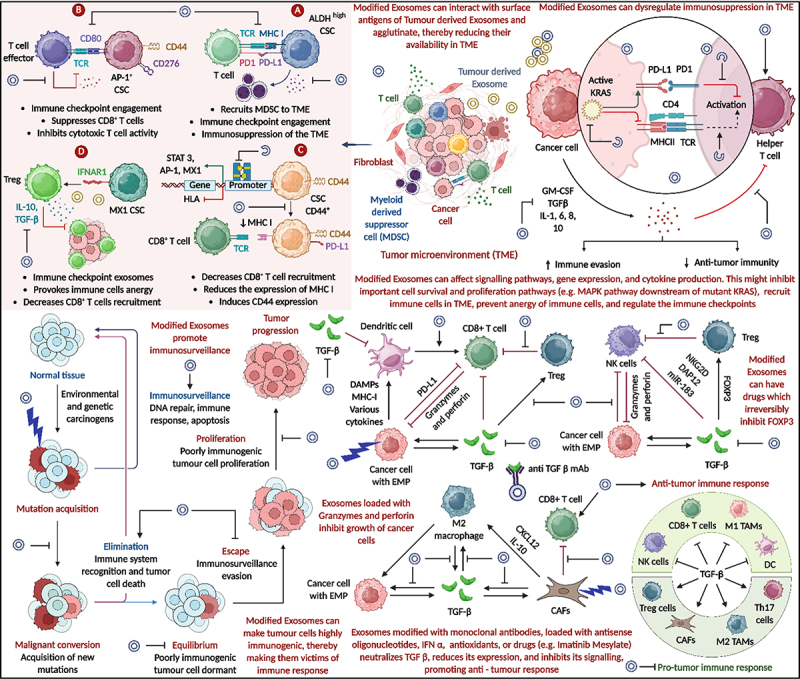
The role of modified exosomes in overcoming tumor-induced immunosuppression through anti-TGFβ activity. Engineered exosomes disrupt TGFβ signaling, reduce regulatory T cell and MDSC recruitment, promote M1 macrophage polarization, and enhance CD8^+^ T cell and NK cell responses. These modifications restore immunosurveillance, inhibit tumor progression, and reprogram the tumor microenvironment to support anti-tumor immunity (created with BioRender.com).

Exosome-based nanovaccines exhibiting anti-TGFβ activity have emerged as a potent approach to enhance antitumor immunity and restore immunosurveillance. TGFβ, a key immunosuppressive cytokine in the TME, promotes tumor progression by inhibiting cytotoxic T cell activation, fostering regulatory T_reg_ function, and facilitating the polarization of macrophages toward the immunosuppressive M2 phenotype. These effects collectively suppress immune-mediated tumor clearance and promote immune evasion. Modified exosomes can be engineered to carry monoclonal antibodies, antisense oligonucleotides, siRNA, or small molecule inhibitors that specifically target and neutralize TGFβ. By inhibiting TGFβ expression or signaling, these exosomes can reduce the recruitment and activity of myeloid-derived suppressor cells (MDSCs), limit the expansion of T_regs_, and shift macrophage polarization toward the M1 (pro-inflammatory) phenotype. Additionally, modified exosomes can carry granzyme- and perforin-loaded cytotoxic payloads, further enhancing the elimination of cancer cells. Moreover, these nanovaccines can interfere with immune checkpoint pathways, such as PD-1/PD-L1, by modulating expression or delivering blocking agents, thereby reinvigorating exhausted CD8+ T cells. Collectively, exosome nanovaccines that block TGFβ signaling not only dismantle immune suppression in the TME but also reprogram it into an immunostimulatory niche, facilitating robust anti-tumor immune responses and enhancing the effectiveness of immunotherapy. Engineered exosomes enhance immunosurveillance, activate anti-tumor responses, and modulate cytokine/chemokine signaling. Functionalization with monoclonal antibodies allows them to bind and sequester TGF-β, reducing it signaling impact (Figure 7).

### Engineered exosomes from dendritic cells in targeted cancer therapy

**Figure 8.**
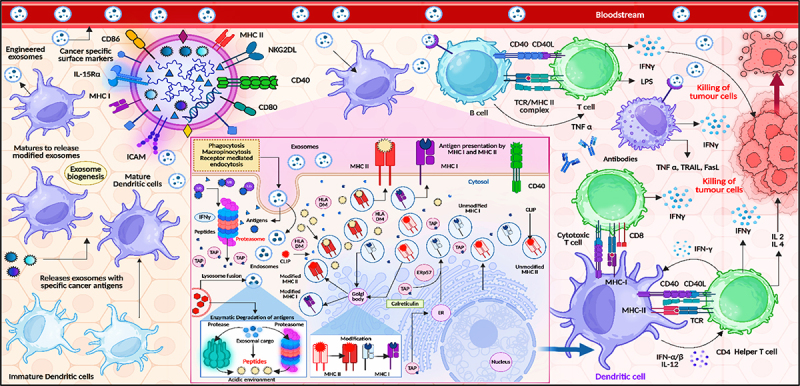
Engineered exosomes from dendritic cells in targeted cancer therapy (created with BioRender.com).

Engineered exosomes derived from dendritic cells (DCs) represent a powerful and precise tool in targeted cancer immunotherapy. These exosomes inherit the antigen-presenting capabilities of their parental DCs, carrying MHC class I and II molecules, co-stimulatory proteins (CD80, CD86, CD40), intercellular adhesion molecules (ICAMs), and tumor-associated antigens (TAAs). As illustrated in Figure 8, immature DCs capture cancer-specific antigens and undergo maturation, a process that triggers exosome biogenesis and the release of antigen-loaded exosomes into the bloodstream. These mature, engineered exosomes are equipped with surface markers like MHC-I, MHC-II, IL-15 Rα, NKG2DL, and co-stimulatory ligands, making them competent in initiating adaptive immune responses. Upon encountering immune cells, these exosomes are internalized via phagocytosis, macropinocytosis, or receptor-mediated endocytosis. Within antigen-presenting cells, exosomal antigens undergo proteasomal and lysosomal processing, followed by peptide loading onto MHC molecules through transporter-associated proteins (TAPs), endosomes, and Golgi-mediated modification. This pathway enables accurate antigen presentation on both MHC I and II complexes, priming CD8^+^ cytotoxic T cells and CD4^+^ helper T cells, respectively. Once activated, CD8^+^ T cells recognize tumor cells presenting the same antigens and induce cytotoxic effects through the release of IFN-γ, TNF-α, TRAIL, and FasL, leading to apoptosis and tumor cell lysis. Simultaneously, CD4^+^ T cells support the immune response by secreting cytokines such as IL-2, IL-4, and IFN-γ, which enhance cytotoxic activity and support memory T cell formation. Furthermore, B cells, upon antigen stimulation via CD40–CD40L and TCR/MHC II interactions, produce tumor-specific antibodies, contributing to antibody-dependent cellular cytotoxicity (ADCC). This multi-tiered immune activation culminates in potent and targeted tumor cell killing, as shown in the upper right of the figure. The strategic use of dendritic cell-derived exosomes, with their natural immunostimulatory features and engineered precision, not only enhances antigen-specific T cell responses but also offers a cell-free vaccine platform with reduced risk of systemic toxicity. Therefore, they represent a robust modality for personalized cancer vaccines and immune-based therapeutic strategies.

### Modified exosomes regulate signaling cascades in MAPK and PPAR pathways

**Figure 9.**
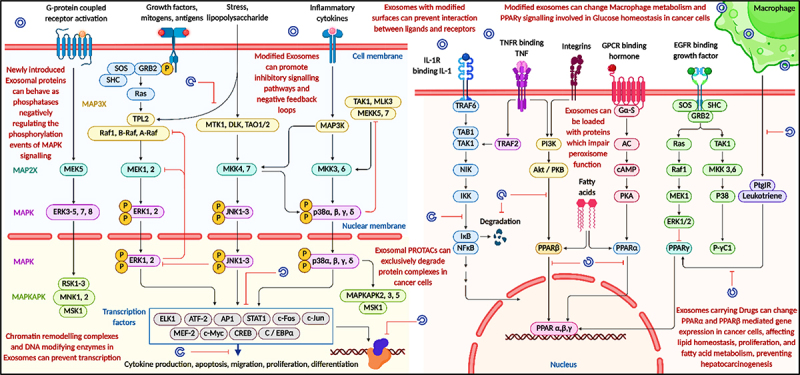
Engineered DC-derived exosomes modulate key cancer-related signaling pathways. Exosome-mediated interference with MAPK, NF-κB, and PPAR pathways, inhibition of transcription factor activation, disruption of receptor-ligand interactions, and reprogramming of macrophage metabolism. These mechanisms collectively contribute to reduced tumor growth, inflammation, and metastasis (created with BioRender.com).

Engineered exosomes derived from DCs are gaining recognition as a highly effective and targeted tool in cancer therapy. These exosomes inherit the antigen-presenting abilities of their parent cells and can be enriched with tumor-associated antigens (TAAs), MHC molecules, and co-stimulatory signals such as CD40, CD80, and CD86. Once administered, they facilitate the activation of cytotoxic T cells and helper T cells, contributing to a robust immune response against cancer cells. However, their therapeutic role goes beyond immune stimulation. These exosomes also interact with and modulate various intracellular pathways that are critical for tumor cell function. They can introduce molecules that interfere with key signaling cascades, including the MAPK, NF-κB, and PPAR pathways. These pathways regulate cell proliferation, survival, inflammation, and metabolism – processes that are often dysregulated in cancer. For example, exosomes may carry molecules that inhibit MEK, ERK, JNK, or p38 MAPK, thereby reducing activation of downstream transcription factors like AP-1, STAT1, and c-Fos, which drive cancer progression. Furthermore, exosomes can affect the behavior of tumor cells by altering receptor-ligand interactions on their surfaces, such as integrins, GPCRs, TNFRs, and growth factor receptors. This disruption hampers the tumor’s ability to respond to external growth cues. Importantly, these exosomes also influence macrophage metabolism and PPAR signaling, which are involved in lipid balance and glucose utilization in the tumor environment. By affecting PPARα, PPARβ, and PPARγ activity, engineered exosomes can reprogram macrophages and impair metabolic support to the tumor, ultimately reducing tumor viability and growth. In summary, dendritic cell-derived exosomes provide a two-pronged therapeutic approach: activating the immune system and directly targeting cancer cell signaling and metabolism. Modified exosomes regulate signaling cascades in MAPK and PPAR pathways. This results in altered cell survival, metabolism, and proliferation dynamics in tumor cells (Figure 9).

### Exosome nanovaccines against EMT

**Figure 10.**
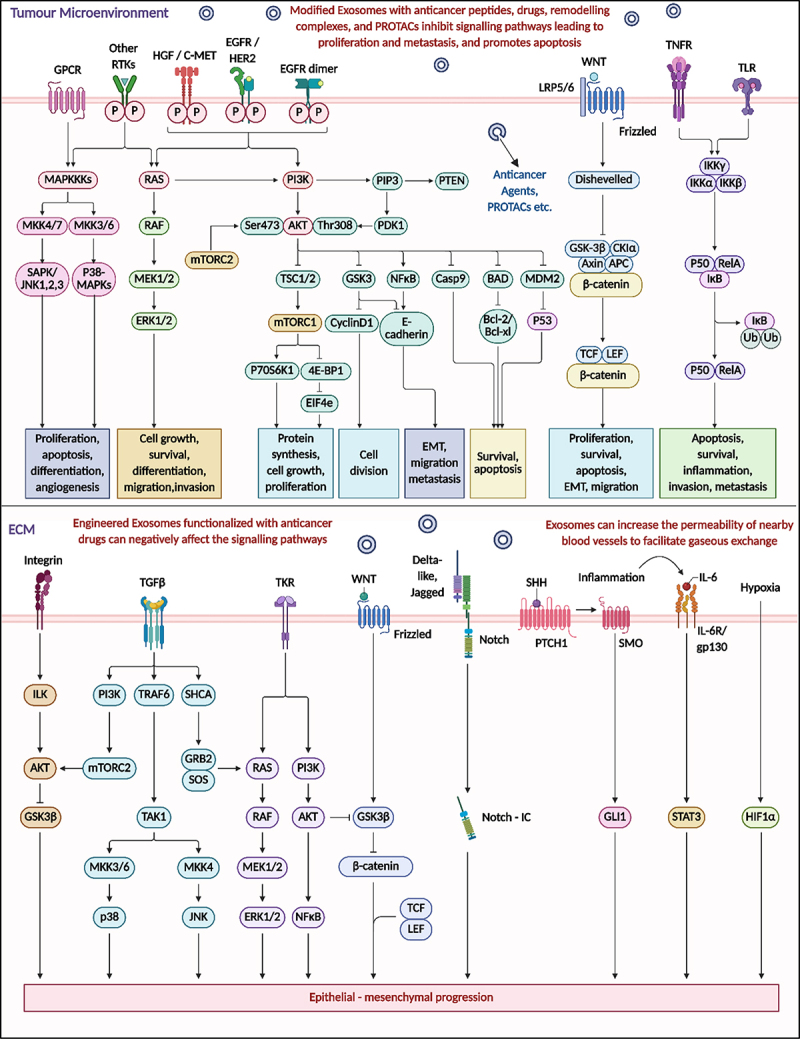
Key signaling pathways involved in EMT within the tumor microenvironment and extracellular matrix. Engineered exosomes, loaded with anticancer agents and signaling inhibitors, can target pathways such as PI3K/AKT, MAPK/ERK, TGF-β, WNT/β-catenin, Notch, and NF-κB to suppress EMT, reduce metastasis, and promote apoptosis in cancer cells (created with BioRender.com).

Exosome nanovaccines offer a cutting-edge and targeted approach to counteract EMT, a critical process in cancer metastasis, immune evasion, and resistance to therapy. EMT involves the loss of epithelial characteristics and the acquisition of a mesenchymal phenotype, enabling tumor cells to migrate, invade, and disseminate. Figure 10 illustrates the complexity of EMT regulation, which is orchestrated by a network of signaling pathways originating from both the TME and the ECM. These include key drivers such as PI3K/AKT/mTOR, MAPK/ERK, TGF-β, WNT/β-catenin, Notch, NF-κB, HGF/c-MET, and hypoxia-induced pathways (e.g., HIF1α and IL-6/STAT3). Engineered exosome nanovaccines can be functionalized with a variety of anti-cancer cargos, including small-molecule inhibitors, PROTACs, siRNAs, and immune-stimulatory peptides, which target multiple nodes of these EMT-related pathways. For example, inhibition of AKT/mTORC1/2 through exosomal delivery downregulates mesenchymal markers and promotes epithelial traits. Suppression of β-catenin via WNT pathway interference can prevent transcription of EMT-promoting genes (e.g., Snail, Slug, and ZEB1). Exosomes targeting the TGF-β/SMAD axis reduce activation of downstream transcription factors driving cell motility and fibrosis. Additionally, by delivering agents that interfere with NF-κB and STAT3, these nanovaccines can inhibit inflammation-induced EMT and restore tumor immunogenicity. Moreover, exosome-mediated restoration of E-cadherin expression and inhibition of matrix metalloproteinases (MMPs) help to maintain epithelial integrity and prevent extracellular matrix degradation, further suppressing metastasis. Notably, exosome nanovaccines can also alter the tumor microenvironment by modulating stromal and immune cells, reducing factors that sustain EMT, such as IL-6, HGF, and hypoxic signaling. By simultaneously targeting multiple signaling axes shown in Figure 10, exosome nanovaccines provide a multi-pronged, systems-level intervention to reverse or inhibit EMT, thus limiting cancer dissemination and improving the efficacy of immunotherapy and chemotherapeutic regimens.

### Exosome nanovaccines as suppliers of specialized cargo for enhanced targeted cancer therapy

**Figure 11.**
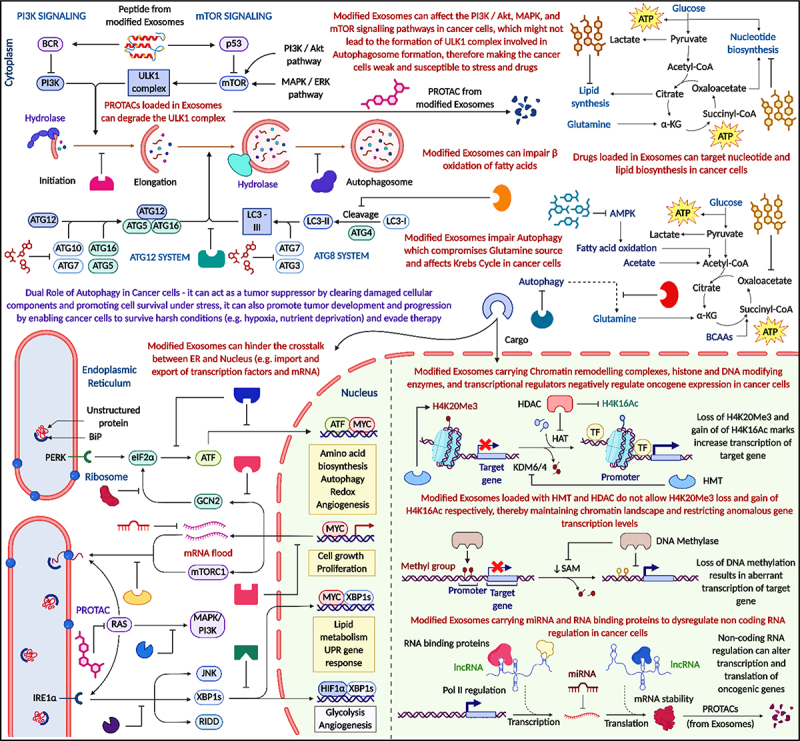
Modified exosomes regulate cancer cell metabolism, autophagy, and gene expression. Exosomal cargo influences key pathways (PI3K/AKT, mTOR, MAPK), disrupts autophagy, impairs lipid and glucose metabolism, and alters chromatin structure through histone modifiers and non-coding RNAs. These mechanisms collectively enhance the therapeutic impact of exosome-based interventions in cancer (created with BioRender.com).

Exosome nanovaccines have emerged as a highly promising modality in targeted cancer therapy, offering a natural and effective means of delivering immunogenic and therapeutic cargo directly to tumor sites. These nanoscale extracellular vesicles can be engineered to carry tumor-associated antigens, immunostimulatory molecules, and adjuvants, thereby enhancing antigen presentation and stimulating a robust, specific immune response. Their intrinsic biocompatibility, prolonged circulation, and ability to cross biological barriers make them particularly suitable for precise, localized therapy with reduced systemic toxicity. Surface functionalization of exosomes with targeting ligands or homing peptides further improves their specificity, allowing for efficient interaction with dendritic cells and activation of T lymphocytes within the tumor microenvironment. Unlike synthetic delivery systems, exosomes benefit from minimal immunogenicity and enhanced uptake by recipient cells due to their endogenous origin. As a result, exosome nanovaccines represent a sophisticated platform for cancer immunotherapy, capable of integrating immune activation with targeted delivery. Figure 11 provides an overview of how modified exosomes influence multiple cancer-associated pathways. These exosomes can modulate signaling cascades such as PI3K/AKT, MAPK/ERK, and mTOR, thereby impacting autophagy, metabolism, and cell survival. PROTACs or hydrolases delivered via exosomes can disrupt the ULK1 complex and autophagosome formation, sensitizing cancer cells to metabolic stress and treatment. Moreover, modified exosomes can impair fatty acid β-oxidation, glutamine metabolism, and lipid biosynthesis – key pathways in tumor cell energy homeostasis. On an epigenetic level, exosomal cargo such as histone modifiers (e.g., HDACs, HATs, KDM6A) and chromatin remodelers can alter transcriptional landscapes in cancer cells. The loss of repressive histone marks (H4K20me3) and gain of activating marks (H4K16ac) promote oncogene expression and disrupt normal gene regulation. Additionally, non-coding RNAs (e.g., lncRNAs, miRNAs) and RNA-binding proteins delivered by exosomes can interfere with transcription, mRNA stability, and translation, further contributing to oncogenic reprogramming. It also emphasizes the dual role of autophagy in cancer – acting as a tumor suppressor in early stages by clearing damaged components, and as a survival mechanism under stress conditions in advanced cancers. Modified exosomes exploit this duality by targeting autophagy-related machinery and metabolic circuits, thereby disrupting cancer cell adaptation and resistance. Collectively, the visual framework in Figure 11 highlights the multifaceted role of engineered exosomes in modulating intracellular signaling, metabolism, and gene expression for enhanced therapeutic efficacy in cancer treatment. Cargo-loaded exosomes influence gene expression, chromatin structure, and protein modification pathways. PROTACs in exosomes can degrade specific proteins (e.g., ULK1), disrupting autophagy and related signaling networks (Figure 11).

Bioinspired exosomes offer several advantages over synthetic nanoparticles due to their innate biocompatibility, low immunogenicity, and cell-specific tropism. These characteristics enhance their potential as therapeutic delivery vehicles by reducing adverse immune responses and improving the precision of payload delivery.^8,41^ Compared to synthetic nanocarriers, bioinspired EVs are more naturally equipped to navigate biological environments, which facilitates improved therapeutic uptake and minimizes systemic toxicity. The intrinsic properties of bioinspired EVs – particularly their immunological compatibility and natural targeting ability – position them as superior candidates for advanced drug delivery applications.^39^

## Cancer detection using exosome-based chipsets

**Figure 12.**
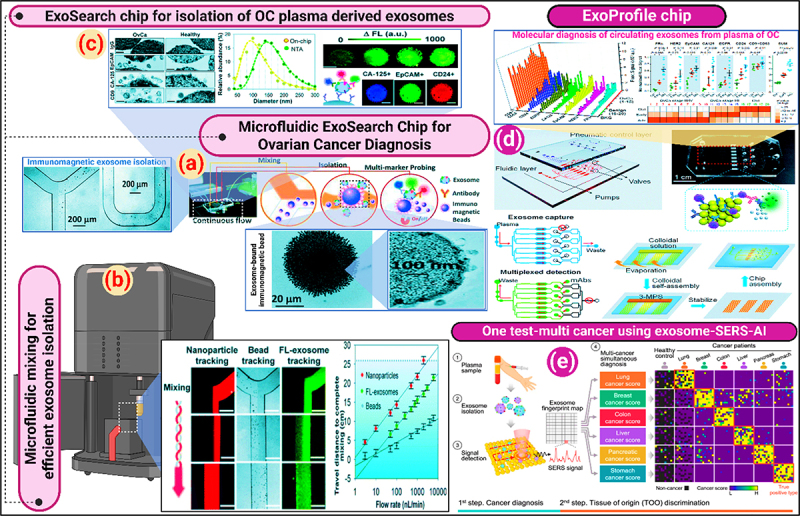
(a) ExoSearch chip for continuous mixing, isolation and in situ, multiplexed detection of circulating exosomes, (b) Microfluidic continuous-flow mixing for efficient exosome isolation, (c) Microfluidic ExoSearch chip for specific isolation of ovarian cancer plasma derived exosomes (reproduced with permission from ref.^51^ copyright @ 2016 the authors); (d) ExoProfile chip for exosome capture for detection (reproduced with permission from ref.^49^ copyright @ 2019 the authors); (e) One test-multi cancer using exosome-SERS-AI (reproduced with permission from ref.^52^ copyright @ 2023 the authors).

Exosome-based chipsets for the detection of cancer is a new application of oncological diagnostic technology. Exosomes represent a natural vehicle for sharing nodes with its cell-of-origin’s molecular signatures and should prove to be valuable in the early detection and monitoring of cancer. Exosomes are nano-sized vesicles that carry molecular messengers of biological signals and travel throughout the body in bodily fluids as a part of the physiological route of blood, vesicles circulate with other particles and proteins, including exosomes that carry protein, lipids, and nucleic acids that can identify molecular signatures which identify the relevant physiological state of the host, including malignant. Exosome analysis to chip technology are pathways to develop sensitive and specific diagnostic tools that require little or no invasive procedures for patients. Exosome-based chipsets permit exosome capture and analysis from biological fluids, such as blood, urine, and saliva. In the case of detecting cancer, chipsets use microfluidics and capture exosomes regardless of the method of isolation or analysis, as the most common methodologies use size exclusion to filter the exosomes, or selectively isolate the exosomes using immunoaffinity, or capture exosomes using microfluidic vortex technology where exosomes are trapped in the microfluidic scale of the hydrodynamic forces that are generated in the system to allow other particles to flow through^50^ (Figure 12). After the exosomes are isolated, they can be analyzed to identify specific cancer-associated biomarkers. The characterization of exosome biomarkers is typically executed using surface plasmon resonance, electrochemical sensors, or surface fluorescence labeling to sample a wide range of indicators of proteins, lipids, and genetic materials expressed in the exosome.^53^

**Figure 13.**
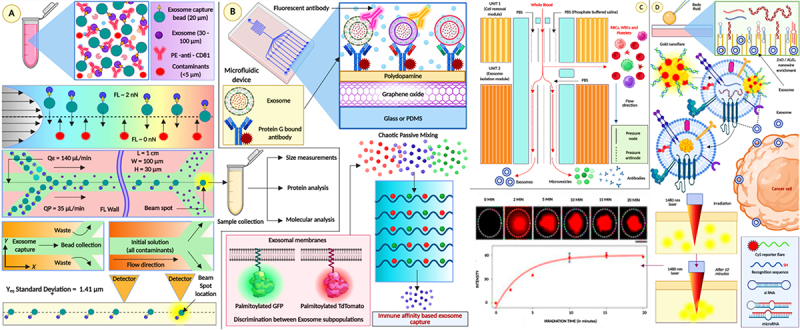
(A) Purification and enrichment of exosomes; (B) Immunoaffinity enrichment of exosomes; (C) Isolation of exosomes from whole blood; (D) Nanowire and nanoflare enrichment of exosomes (reproduced with permission from ref.^57^ copyright @ 2020 the authors).

Recent developments in exosome-based chipsets have concentrated on heightening detection sensitivity and specificity. Many researchers have developed chipsets to create nanostructured substrates to increase the area for exosome binding and interactions. This alone can dramatically enhance detection limits for cancer biomarkers.^54^ Furthermore, advancements in computational methods and machine learning algorithms for use with chipset technology will enhance the analysis of complex data from exosome biomarkers. As a result, researchers will gain a more robust ability to discriminate between benign conditions and malignant states based on exosome profiles.^55^ The potential adverse implications of exosome-based chipsets in clinical practice are significant, and if, exosome-based chipsets provide a tool for early cancer detection, these devices could substantially alter patient outcomes with faster intervention. Furthermore, the meaningful implications of a noninvasive sample collection for exosome analysis are the ease of use for routine monitoring, offering a clear opportunity to assess the tumor’s dynamic change and treatment effectiveness in “real-time”^56^ (Figure 13). Exosomes, compared to ctDNA and CTCs, are more stable, capture genomic and proteomic characteristics of the tumor, and provide earlier signals. ctDNA has an advantage in specificity for genomic mutations. A limitation of CTCs is their scarcity in early-stage cancers.

For the future, the difficulty lies in creating uniform techniques for isolating and analyzing exosomes so that consistent, reliable results can be obtained across different clinical environments. Additionally, researchers are working on how to utilize this technology at scale in order for it to be common as a diagnostic tool in oncology.^58^ In addition to diagnostics, exosomes also provide a pathway toward treatment as part of nano vaccines format. Researchers could utilize exosomes’ innate ability to carry “cargo,” and design vaccines whereby tumor antigens are specifically delivered to immune cells, thus augmenting the immune system’s ability to identify and destroy cancer cells.^59^ The process of exosome nano vaccine creation can involve isolating exosomes from tumor cells or dendritic cells with tumor antigens. Then, specific cancer antigens can be loaded on exosomes and then provided to the patient, as they interact with dendritic cells, notifying the body to induce a specific immune response.^60^ Exosome chipsets do not only change cancer detection from a diagnostic standpoint and provide the ability for earlier, noninvasive, and precise diagnostic testing, but bridge the gap to therapeutic applications, like nano vaccines. As research moves forward, exosome diagnostics joined with therapeutics should lend itself to a comprehensive method of managing cancer and improving outcomes in oncology.^61^

## Challenges and future directions

Pursuing an effective exosome-based cancer vaccine presents numerous major issues. Developing effective vaccines has to consider the many heterogeneous aspects of tumors. The heterogeneity seen in tumors makes finding reliable biomarkers an arduous task and alters the consistency of vaccine preparation which translates into variations in vaccine effectiveness. Tumor heterogeneity particularly creates a problem since it affects standardizing treatment between patients for clinically widespread application.^62^ Additionally, the technical challenges of isolating and characterizing an exosome of high purity and high specificity have their own challenges as it relates to development and application of cancer vaccines. The previous golden standards of exosome isolation, such as ultracentrifugation, and size exclusion chromatography, though able to isolate, may not effective to use or may still have contaminating matter that does not get removed. Additionally, identify challenges with using new technologies like microfluidics and immunoaffinity based technology, in terms of efficiency, scalability, and reproducibility.^63^ Furthermore, in the vaccination community there are no standardized protocols for exosome isolation or quantification. Neither any standard meaning reproducible protocols, affect experimental reproducibility, as any variability will alter research findings and complicate regulatory approvals, adding to the challenges from lab to clinic.^64^ In order to satisfy the clinical requirements, the next challenge is to enhance production while preserving the biological activity and biophysical characteristics of the exosomes. This is an important issue because the focus will be on developing standard, reproducible, and efficient production systems that can operate at an appropriate scale.^65^ Additionally, the interaction with the immune system is a complex balance to achieve appropriately. To develop a proper antitumor immune response with an exosome-based vaccine, the tumor antigens should be effectively presented to the immune cells, while avoiding immune tolerance or unwanted adverse reactions, and ultimately compromising treatment.^28,49,66–74^

Looking forward, improving diagnostic and monitoring tools to profile exosome profiles accurately, could change the landscape for early detection and monitoring of cancer treatment responses.^67^ There is also great potential for advancements in the targeted delivery systems. By engineering exosomes to express specific ligands or antibodies we could increase the uptake of exosomes by the target cells and improve efficacy and specificity^68^ of the vaccine. Further work on the ability to personalize exosome therapies has significant promise for improving outcomes. Developing strategies for tailoring therapies to the specific tumor profile of each patient would make these treatments much more effective with less morbidity.^69^ In order to overcome these challenges, collaborative and interdisciplinary science will be essential. By integrating current knowledge and approaches in oncology, immunology, nanotechnology, and bioengineering, we can accelerate the process for application of basic science to therapies that could revolutionize the way we treat cancer, advancing toward more personalized, efficacious, and less invasive treatments.^70^ There are numerous translational barriers to overcome including scalable GMP supply, batch-to-batch consistency, and regulation. These barriers need addressed prior to successful commercialization of EV based vaccines.^36^

## Conclusion

This review integrates and extends the findings of Batista^75^, Youssef et al.^76^, and Wang et al.^77^, providing a comprehensive and systematic comparison of therapeutic approaches, loading strategies, and translational challenges in the context of exosome-based cancer vaccines.^75–77^ Unlike Tang et al.^78^, who focus solely on exosome-based liquid biopsy platforms, this review uniquely emphasizes both diagnostic and therapeutic dimensions.^78^ The development of exosome-based cancer vaccines represents a promising frontier in oncology, capitalizing on the natural targeting capabilities and bioavailability of exosomes to facilitate precise immunotherapeutic interventions.^36,79^ These vesicles offer an innovative mechanism for delivering tumor antigens, but their clinical translation is impeded by several barriers, including tumor heterogeneity, lack of standardized isolation protocols, and difficulties in ensuring batch-to-batch consistency.^80^ Moreover, regulatory uncertainties and scalability issues continue to pose significant hurdles.^81^ Overcoming these challenges requires the integration of nanotechnology, molecular engineering, and systems biology. Recent advances in these areas provide a hopeful outlook for future application.^41^

As we further dissect the interactions between exosomes and the tumor microenvironment, interdisciplinary collaboration will be vital. Establishing standardized protocols, investing in scalable production technologies, and refining targeting mechanisms will pave the way for more personalized, efficient, and accessible cancer treatment options. Exosome-based vaccines, once fully realized, have the potential to significantly redesign the landscape of oncology and immunotherapy.

## Data Availability

No new data was generated in the current review
